# Organic fertilizer application promotes the soil nitrogen cycle and plant starch and sucrose metabolism to improve the yield of *Pinellia ternata*

**DOI:** 10.1038/s41598-024-63564-0

**Published:** 2024-06-03

**Authors:** Lu Wei, Jinxin Li, Kaili Qu, Hong Chen, Mingxing Wang, Shuaijie Xia, Huixia Cai, Xi-En Long, Yuhuan Miao, Dahui Liu

**Affiliations:** 1 Pharmacy Faculty, Hubei University of Chinese Medicine, Wuhan, 430065 China; 2 School of Geographic Sciences, Nantong University, Nantong, 226019 Jiangsu China

**Keywords:** Organic fertilizer, *Pinellia ternata*, Yield, Microbial community, Nitrogen cycle, Transcriptome, Agroecology, Ecosystem ecology

## Abstract

*Pinellia ternata* (Thunb.) Breit is a traditional Chinese medicine with important pharmacological effects. However, its cultivation is challenged by soil degradation following excessive use of chemical fertilizer. We conducted an experiment exploring the effects of replacing chemical fertilizers with organic fertilizers (OF) on the growth and yield of *P. ternata*, as well as on the soil physicochemical properties and microbial community composition using containerized plants. Six fertilization treatments were evaluated, including control (CK), chemical fertilizer (CF), different proportions of replacing chemical fertilizer with organic fertilizer (OM_1−4_). Containerized *P. ternata* plants in each OF treatment had greater growth and yield than the CK and CF treatments while maintaining alkaloid content. The OM_3_ treatment had the greatest yield among all treatments, with an increase of 42.35% and 44.93% compared to the CK and CF treatments, respectively. OF treatments improved soil quality and fertility by enhancing the activities of soil urease (S-UE) and sucrase (S-SC) enzymes while increasing soil organic matter and trace mineral elements. OF treatments increased bacterial abundance and changed soil community structure. In comparison to the CK microbial groups enriched in OM_3_ were *OLB13*, *Vicinamibacteraceae*, and *Blrii41*. There were also changes in the abundance of gene transcripts among treatments. The abundance of genes involved in the nitrogen cycle in the OM_3_ has increased, specifically promoting the transformation of N-NO_3_^−^ into N-NH_4_^+^, a type of nitrogen more easily absorbed by *P. ternata*. Also, genes involved in "starch and sucrose metabolism" and "plant hormone signal transduction" pathways were positively correlated to *P. ternata* yield and were upregulated in the OM_3_ treatment. Overall, OF in *P. ternata* cultivation is a feasible practice in advancing sustainable agriculture and is potentially profitable in commercial production.

## Introduction

Soil fertility is the foundation of economical and efficient crop production. Soil can continuously supply necessary water, nutrients, and other factors needed for plant growth and development^1^. However, long-term crop production and monoculture can lead to a decrease in soil fertility, resulting in reduced crop yields^2,3^. To avoid reduced yields, intensive agricultural management systems have been developed with a focus on using fertilizers to help meet the growing population's demand for food crops and herbs^4,5^. While the extensive use of chemical fertilizers can help to maintain high crop yields^6^, chemical fertilizers can also lead to serious problems such as soil hardening, acidification, and decreased organic matter and soil fertility^7^. Fortunately, organic fertilizers can increase soil nutrients, organic matter content, enzyme activity^8^, and microbial abundance^9^, and are generally considered a suitable substitute for chemical fertilizers.

Soil microbes play a critical role in soil organic matter decomposition and nutrient cycling in ecosystems and are the most sensitive indicators of soil quality^10^. The community structure and function of soil microorganisms can quickly shift in response to changes in agricultural practices or the environment, altering the nutrient utilization efficiency and soil productivity in the agricultural field^11^. Organic fertilizers are rich in organic matter and may contain beneficial microorganisms that can improve soil physical and chemical properties, change microbial community composition, and promote beneficial microorganism enrichment, ultimately positively impacting crop growth and yield. A previously published 2-year field trial indicated that organic fertilizers can enrich specific microorganisms associated with organic matter degradation, such as *Chloroflexi*, *Acidobacteria*, and *Basidiomycota*, which increased the availability of soil nutrients and ultimately increased pineapple yield^12^. Pereg et al. (2018) have shown that soil microbial communities involved in the biological nitrogen cycle (especially nitrogen fixation and denitrification) under organic fertilizer management were more abundant. This enhanced nitrogen cycle helped to maintain soil health and increased grape yields^13^. However, changes in soil microbes are closely related to soil enzyme activity variation, as soil enzymes play a crucial role in soil nutrient mineralization, microbial metabolism and plant nutrient cycling^14^. Among them, soil urease, acid phosphatase and sucrase can hydrolyze macromolecular substances such as polysaccharide and proteins, forming small molecular substances that are easily absorbed by plants^15^. The activity of catalase can indicate the degree of organic matter accumulation^16^. These enzymes play a necessary role in soil material cycling and energy flow. In rice cultivation, the application of organic fertilizer can enhance soil urease and catalase activities, further enrich beneficial bacteria such as *Bacillus* and *Flavobacterium*, thus promotes the improvement of rice annual yield^17^. Therefore, observing the changes in soil enzyme activity, microbial composition and function may provide key insights into the mechanisms behind the effects of organic fertilizer application on soil improvement and plant growth.

*Pinellia ternata* (Thunb.) Breit is an herb of the *Araceae* family and is widely distributed in China, where it has a long history of being processed into medicine. Its dried tuber has traditionally been considered to have important pharmacological properties, including resolving phlegm and stopping vomiting^18^. Modern pharmacological studies also have found that *P. ternata* has many pharmacological properties, such as anti-tumor efficacy^19^, lowering blood lipids, and treating coronary heart disease^20^. Although *P. ternata* is widely distributed, due to increasing demand and the deterioration of ecosystems in recent years, wild *P. ternata* populations have sharply decreased^21^. Agricultural production of *P. ternata* has become more common in order to meet the demand for this herb^22^. Herb farmers are continuously expanding the cultivation of *P. ternata* under the conventional chemical agriculture model in order to increase yields, which is often accompanied by serious problems such as degradation of *P. ternata* quality, aggravation of diseases and pests, and accumulation of pesticide residues and heavy metals^23^.

"Traditional Chinese medicine (TCM) ecological agriculture cultivation" was proposed in China in 2015, to encourage eco-friendly practices such as reducing the use of chemical fertilizers and pesticides^24^. The application of TCM ecological agriculture cultivation practices has been explored for *P. ternata*. For example, several microbial agents, such as *Streptomyces jingyangensis* T. and *Bacillus mucilaginosus* A., have been used in *P. ternata* cultivation to improve the community structure of soil microorganisms, increase tuber yield, and reduce plant mortality^25^. In addition, it was found that intercropping *P. ternata* with soybeans would produce high yields and that intercropping with pepper would increase soluble sugar, succinic acid, and guanosine content^26^. Moreover, based on the ideal growing conditions of *P. ternata*, an ecologically based producing strategy known as “Harvest Every Year with One Sowing” has been formulated to reduce the impact of *P. ternata* cultivation^27^.

Overall, the soil factors leading to yield reduction and increased disease pressure in *P. ternata* cultivation mainly include the deterioration of soil physical and chemical properties, nutrients imbalance, and detrimental changes in the microflora community^10,28,29^. A healthy soil environment is a prerequisite for normal plant growth, and current ecological planting measures mainly focus on improving soil nutrient status, enzyme activity, and microbial species and functions, enhancing the yield and ensuring the quality. Despite the improvements in the sustainable production of *P. ternata*, the exploration of reducing chemical fertilizers in favor of organic fertilizers has been ignored. Chemical fertilizer has the characteristics of high concentration and fast release speed. On the contrary, organic fertilizers have relatively low fertility and slower release rates. These properties mean that organic fertilizer often cannot meet the demand of crop growth over short timeframes^9^. Additionally, heavy applications of organic fertilizers may cause competition for nutrients between crops and soil microorganisms that decompose fertilizers^11^. Therefore, replacing chemical fertilizers with organic fertilizers (OF) as a beneficial practice to overcome this problem has been vigorously promoted in recent years, with adaption by commercial producers across several crops^30–32^.

OF have also been successfully used in the cultivation of some TCM materials. Pacheco et al. (2021)^33^ found that the use of animal manure in the organic cultivation of *Passiflora incarnata* can increase phenolic compounds in leaves and the antioxidant activity of the plant, thereby improving its medicinal quality. In systems developed for the high-yield and high-quality culture of *Perilla frutescens* (Linn.) Britt*.*, an OF ratio of 50% promoted the accumulation of antioxidant components in the plants and increased plant biomass and grain yield^32^. However, different plant materials require different fertilization strategies, so the proportion of chemical fertilizers that organic fertilizers should replace may differ greatly between crops. The application of OF in *P. ternata* cultivation to improve yield and quality while reducing negative environmental effects deserves further exploration.

In this study, we hypothesized that OF could improve soil nutrients, increase soil enzyme activity, augment the microbial availability, and increase *P. ternata* yield. So, a containerized plant experiment was conducted to investigate the effect of OF on soil properties, *P. ternata* growth and yield under balanced fertilization regimes. Furthermore, microbial diversity analysis, metagenome sequencing, and transcriptome sequencing were conducted to explore the mechanisms behind OF effects on *P. ternata* from both the soil and plant perspectives. The specific purpose of this study is comparing the effects of OF treatments on the growth and yield of *P. ternata*, and determine the optimal fertilization ratio for *P. ternata* cultivation, revealing the changes in soil environment, microbial community and function after OF treatment; analyzing the molecular mechanism of *P. ternata* responding to OF treatment and achieving yield increase. This work will optimize the productivity of *P. ternata* while minimizing the environmental impact of chemical fertilizers and will provide theoretical support for *P. ternata* cultivation.

## Materials and methods

### Materials

The *P. ternata* cultivar 'Qian Banxia' was obtained from Qianjiang Qianbanxia Pharmaceutical Co., Ltd. (Hubei, China) in November 2021 for use in this study. Obtained tubers had a tuber diameter between 1.0 and 1.4 cm and hundred-grain weight is about 110 g. Organic fertilizer (N ≥ 2%, P_2_O_5_ ≥ 2%, K_2_O ≥ 2%) was obtained from Hubei Lvdao Agricultural Development Co., LTD. (Hubei, China), and chemical fertilizer (N ≥ 15%, P_2_O_5_ ≥ 15%, K_2_O ≥ 15%) was provided by Sinochem Agro-Ecological Technology Co., LTD (Hubei, China).

The soil media used in horticultural pots was a blended mix of yellow–brown soil, vermiculite, and perlite (v:v:v, 3:2:1). The yellow–brown soil was collected from the medicinal botanical garden of Hubei University of Chinese Medicine (114°15′50″E, 30°27′6″N), Hubei Province. It was a viscous slightly acidic soil, and the total of N, P, and K are 1.07, 0.77, and 22.37 g/kg, respectively. The more information of soil nutrient elements was shown in Table S1. Vermiculite and perlite are beneficial for soil loosening, ventilation, maintaining humidity, and providing growth support.

### Experimental design

The experiment was conducted using containerized plants in the medicinal botanical garden of the Hubei University of Chinese Medicine between November 2021 and May 2022. The region has a subtropical monsoon climate with abundant rainfall and sufficient sunshine. The mean annual precipitation is 1256 mm, the mean annual sunshine is 2076 h, and the mean annual temperature is 21 °C.

To explore the effects of OF on *P. ternata* and the mechanisms behind observed changes, an experiment consisting of six groups (CK: control, CF: chemical fertilizers, OM_1_-OM_4_: OF at different proportions) was conducted. Specifically, the total applied amount of N, P, and K in fertilizers was maintained across different treatments (except CK, which contained control), but five different ratios of chemical fertilizer to organic fertilizer were used across treatments (Table 1). A completely randomized design with five replicates was used.

**Table 1:** The amount of fertilizer applied for each treatment.

Treatments	Organic fertilizers (g/pot)	Chemical fertilizers (g/pot)			Organic fertilizers replacement rate (%)
	Base fertilizer	Base fertilizer	Plant establishment fertilizer	At-flower fertilizer	
CK	0	0	0	0	–
CF	0	6.4	2.13	2.13	0
OM_1_	20	4.8	1.6	1.6	25
OM_2_	40	3.2	1.06	1.06	50
OM_3_	60	1.6	0.53	0.53	75
OM_4_	80	0	0	0	100

CK: no fertilizer, CF: chemical fertilizers, OM_1_-OM_4_: Replacing chemical fertilizers with organic fertilizers in different proportions. The total amount of nutrients in each treatment is equal except in the CK treatment. The proportion of organic fertilizer replacement was calculated according to the amount of N, P, and K in the fertilizers.

In December 2021, each container (47 × 23 × 17.5 cm) was filled with 8 kg of the described soil mix as a replicate. There are a total of 30 containers from six treatment groups with five replicates. The *P. ternata* seed tubers were sterilized with 12.5 mg/L ethyl allicin and 200 mg/L sodium dichloroisocyanurate to reduce disease incidence. In detail, soaked thoroughly for 0.5 h, drained for 10–30 min, then sowed in the pots 5 cm deep with a density of 24 tubers/pot. The between-plant and between-row spacing was 4 cm and 11 cm, respectively. Organic fertilizers were applied prior to sowing (base fertilizer), while chemical fertilizers were split-applied as 60% base fertilizers, 20% additional fertilizer at plant establishment, and 20% additional fertilizer at the flowering stage. During the experiment, cultural management, such as watering and weeding, was consistent among all pots, including the CK replicates.

### Investigation of agronomic traits

*P. ternata* agronomic traits were measured at the flowering stage (May 6 2022). Five randomly selected plants from each pot were measured to determine plant height, petiole length, length and width of leaves, and petiole thickness. Furthermore, soil and plant analyzer development (SPAD) index was determined on leaves with a chlorophyll analyzer (TYS-4N, China). Due to the trifoliate leaf structure of *P. ternata*, all leaf indicators were uniformly measured using the middle leaflet. The measurements from each of the five plants per pot were averaged together, and their mean was used as the value of the respective experimental replicate.

### Measurement of *P. ternata* yield and quality

After the above-ground plant parts were allowed to dry over the summer, all *P. ternata* corms and tubers were carefully harvested on May 30, 2022. After thorough washing with tap water and being allowed to air dry, the corm and tuber biomass were weighed to determine yield. The number and diameter of *P. ternata* corms and tubers were separately quantified to evaluate the yield composition.

As an indicator of quality, alkaloids are considered the main active ingredient of *P. ternata*, and are closely related to the quality of *P. ternata* processed into medicine^34^. Tubers were peeled and dried at 55 °C, then ground into a 65-mesh powder. Then, the total alkaloid content was determined by ultraviolet spectrophotometry as previously described^35^. Specifically, accurately weigh 0.2 g *P. ternata* tuber powder, add 1 mL concentrated ammonia and 10 mL chloroform successively, then soak in ice water for 3 h, conduct ultrasonic extraction for 40 min, and filter at last. The residue was washed in 10 mL chloroform for 3 times (4, 3, 3 mL), the filtrate and lotion were combined. Chloroform was recovered at 60 ℃ until dry, 10 mL of chloroform was added to dissolve and transferred to separation funnel. Add 5 mL of citric acid-sodium citrate buffer (pH = 6), 1 mL of 0.05% bromothymol blue standard solution, and 10 mL of chloroform to the separating funnel in sequence. After full shaking and standing for 30 min, the chloroform layer was separated. The water layer was extracted with chloroform (10 mL) twice in the same way. The chloroform layer was merged, evaporated and recovered to dryness by rotary evaporation at 60 ℃, and then dissolved with chloroform to 10 mL. The absorbance was determined at the wavelength of 416 nm. Using ephedrine hydrochloride as the control substance, the linear equation obtained by the same method with ephedrine hydrochloride as the standard is Y = 0.0049X + 0.1588, R^2^ = 0.9911, and the range is 21.28–106.4 μg/mL.

### RNA extraction, RNA-seq, and DEGs analysis

At harvest, fresh tubers were randomly selected, frozen in liquid nitrogen, ground, and mixed evenly. The control (CK), chemical fertilizer treatment in traditional agriculture (CF), and OF treatment with the highest yield in the study (OM_3_) were selected as representative for RNA-seq. The total RNA from tubers were extracted using the EASY spin Plant RNA Extraction kit (Aidlab, China) according to the manufacturer's guidelines. The sequencing was performed on an Illumina Hiseq 2500 sequencing platform by Shanghai Persenal Biotechnology Co., Ltd. (Shanghai, China). Then, contaminated adapters, poly-N, and low-quality reads (Q < 20) were filtered out, and clean reads from each sample were de novo assembled using Trinity^36^.

The obtained unigenes were annotated according to several publicly available protein databases, including the NR (NCBI non-redundant protein sequences), GO (Gene Ontology), KEGG (Kyoto Encyclopedia of Genes and Genomes), eggnog (Evolutionary Genealogy of Genes: Non-supervised Orthologous Groups), Pfam (Protein family), and the Swiss-Prot protein databases to compare the corresponding function annotations^37^. The unigene expression levels were calculated by RSEM software (v 1.2.15)^38^. The differentially expressed genes (DEGs) were detected using the DESeq package, and significance was determined at an alpha level of 0.05^39^. After screening, differentially expressed genes (DEGs) with log2FoldChange ≥ 1 and *P* < 0.05 were selected for further investigation. Using the default parameters, KEGG enrichment analysis was conducted using the Persenal online analysis website (https://www.genescloud.cn/chart/KEGGenrich). Raw transcriptomic data have been deposited in the NCBI SRA database (BioProject ID: PRJNA1002642.).

### Soil sampling

Soil was sampled from a depth of 5–10 cm from each pot. Sample sites were selected to be near tubers, and collection occurred after harvest (May 30, 2022). From fresh soil samples, plant tissue was removed and placed in sterile centrifuge tubes. Then, the samples were immediately placed in an ice box and transported to the laboratory. Each composite soil sample was divided into two sub-samples, one of which was stored at − 80 °C until DNA extraction. The other sub-sample was air-dried for 1 week, crushed and screened to 100-mesh, and was used for the measurement of physicochemical properties determination.

### Estimation of soil enzymatic activities

Soil urease (S-UE) activity was determined with the S-UE Assay Kit (Beijing Solarbio Science & Technology Co., Ltd.) using the indophenol blue colorimetry method at 630 nm. Soil catalase (S-CAT) activity was measured at 240 nm using the S-CAT Assay Kit (Beijing Solarbio Science & Technology Co., Ltd.). Soil alkaline phosphatase (S-AKP) activity was measured using the S-AKP Assay Kit (Beijing Solarbio Science & Technology Co., Ltd.) with the phenylene disodium phosphate method at 660 nm. Soil sucrase (S-SC) activity was measured using the S-SC Assay Kit (Beijing Solarbio Science & Technology Co., Ltd.) with the 3, 5-dinitrosalicylic acid chromogenic method at 540 nm.

### Determination of soil physicochemical properties

Soil pH was measured using a pH meter (Sartorius PB-10, Germany) in a soil suspension with a soil-to-water ratio of 1:2.5 m/v. The soil organic matter (SOM) content was determined using the potassium dichromate external heating method^40^. Soil alkali-hydrolyzed nitrogen (AN) was quantified using the alkali-hydrolyzed diffusion method^41^. Soil-available phosphorus (AP) was extracted with sodium bicarbonate and analyzed using the molybdenum-antimony resistance colorimetric method^42^. Soil-available potassium (AK) was extracted with ammonium acetate and measured by ultraviolet spectrophotometer^43^. Soil exchangeable calcium (Ca) and magnesium (Mg) were extracted with ammonium acetate and estimated by atomic absorption spectrophotometer^41^. Soil available iron (Fe), manganese (Mn), and zinc (Zn) were determined by ethylene triamine pentetic acid (DTPA) extraction and atomic absorption spectrophotometry^41^.

### Soil DNA extraction

Microbial community genomic DNA was extracted from soil samples using FastDNA^®^ Spin Kit for Soil (MP Biomedicals, Norcross, GA, U.S.) according to the manufacturer’s instructions. The DNA extract was checked on 1% agarose gel, and DNA concentration and purity were determined with a NanoDrop 2000 UV–Vis spectrophotometer (Thermo Scientific, Wilmington, USA).

### 16S rRNA sequencing

The hypervariable region V3–V4 of the bacterial 16S rRNA gene was amplified with the primer pair 338F (5′-ACTCCTACGGGAGGCAGCAG-3′) and 806R (5′-GGACTACHVGGGTWTCTAAT-3′) by an ABI GeneAmp 9700 PCR thermocycler (ABI, CA, USA). The PCR product was checked on 2% agarose gel, then extracted and purified using the AxyPrep DNA Gel Extraction Kit (Axygen Biosciences, Union City, CA, USA) according to the manufacturer’s instructions. Then, the purified amplicons were sequenced on an Illumina Miseq platform at Shanghai Majorbio Bio-pharm Technology Co., Ltd. (Shanghai, China).

The raw data from the 16S rRNA gene sequencing was quality-checked using FASTQ (v 0.20.0)^44^, and OTUs were clustered with a 97% identity cut-off using UPARSE^45^. In order to obtain species classification information corresponding to each OTU, each sequence was annotated by using RDP classifier (v 2.2)^46^, and then was compared with Silva 16S rRNA database (v138). Using Chao and Shannon indexes demonstrated the bacterial community richness and diversity by using Mothur (v 1.30.1)^47^. Principal Coordinates Analysis (PCoA) analyses was operated using QIIME (version 1.9.1) based on unweighted UniFrac distance matrix or Bray–Curtis dissimilarity. Linear discriminant analysis (LDA) coupled with effect size measurements (LEfSe) analysis was completed on the online platform Majorbio Cloud Platform (http://www.majorbio.com), in which the threshold of LDA discriminant analysis of soil bacteria was 4. The raw reads were deposited into the NCBI Sequence Read Archive (SRA) database (Accession Number: PRJNA1012746).

### Metagenomic sequencing

The DNA was fragmented to approximately 400 bp with a Covaris M220 ultrasonicator, and the paired-end library was constructed with the NEXTFLEX Rapid DNA-Seq Kit. The paired-end sequencing was performed using the Illumina NovaSeq Kit on an Illumina NovaSeq platform at Majorbio Bio-pharm Technology Co., Ltd. (Shanghai, China.) per the manufacturer’s instructions. The metagenome sequences have been deposited in the NCBI database under the accession number PRJNA1015345.

The raw data from the metagenomic sequencing were filtered by shear quality (the adapter sequence reads 3’ end and 5’ end), then removing the low-quality reads (length less than 50 bp), reads with ambiguous base “N”, and reads with a mean base quality less than 20 using the fastp software (v 0.20.0)^44^. Then, MEGAHIT (v 1.1.2)^48^ software was used to assemble the optimized sequences, and contigs ≥ 300 bp were screened for downstream analysis. The prediction of open reading frames (ORFs) contigs in concatenation results was performed using Prodigal^49^. The ORFs with nucleic acid length greater than or equal to 100 bp were selected to be translated into amino acid sequences, and CD-HIT (v 4.6.1)^50^ software was used for clustering. The longest gene of each class was selected as the representative sequence to construct a non-redundant gene set. The high quality of every sample read was compared with non-redundant gene sets using SOAPaligner software (v 2.21)^51^, and statistical gene abundance information was calculated using Diamond (v 0.8.35)^52^. The amino acid sequences of the non-redundant gene sets were compared with the NR and KEGG database (the expected value of the BLASTP comparison parameter set was 1 * 10^–5^)^53^. The species annotations were obtained through the taxonomic information database corresponding to the NR database, and then the abundance of the species was calculated using the sum of the corresponding gene abundance of the species. The sum of gene abundances corresponding to KO, pathway, EC, and module was used to calculate the abundance of corresponding functional categories using the KEGG database. The bioinformatics analysis was analyzed on the Majorbio Cloud Platform as described above.

### Statistical analysis

The data were recorded and processed using GraphPad Prism 8 and SPSS 24.0 software. Results were expressed as mean ± standard deviation (SD). The significant differences between different treatments groups were analyzed by One-way analysis of variance (ANOVA), and Least Significant Difference (LSD) post test was applied to further differentiate the mean values at *P* < 0.05. In addition, principal coordinate analysis (PCoA) was used to assess the differences in improvement measures based on Bray–Curtis distance at OUT level. The canonical correspondence analysis (CCA) was used to determine the relationships among the microbial communities, soil properties and soil enzyme activities. The correlation between yield factors and key DEGs of different treatments was calculated by Spearman Rank correlation.

### Ethical approval

All the plant experiments/protocols were performed with relevant institutional, national, and international guidelines and legislation.

## Results

### Replacing chemical fertilizers with organic fertilizers promotes *P. ternata* growth

**Figure 1:**
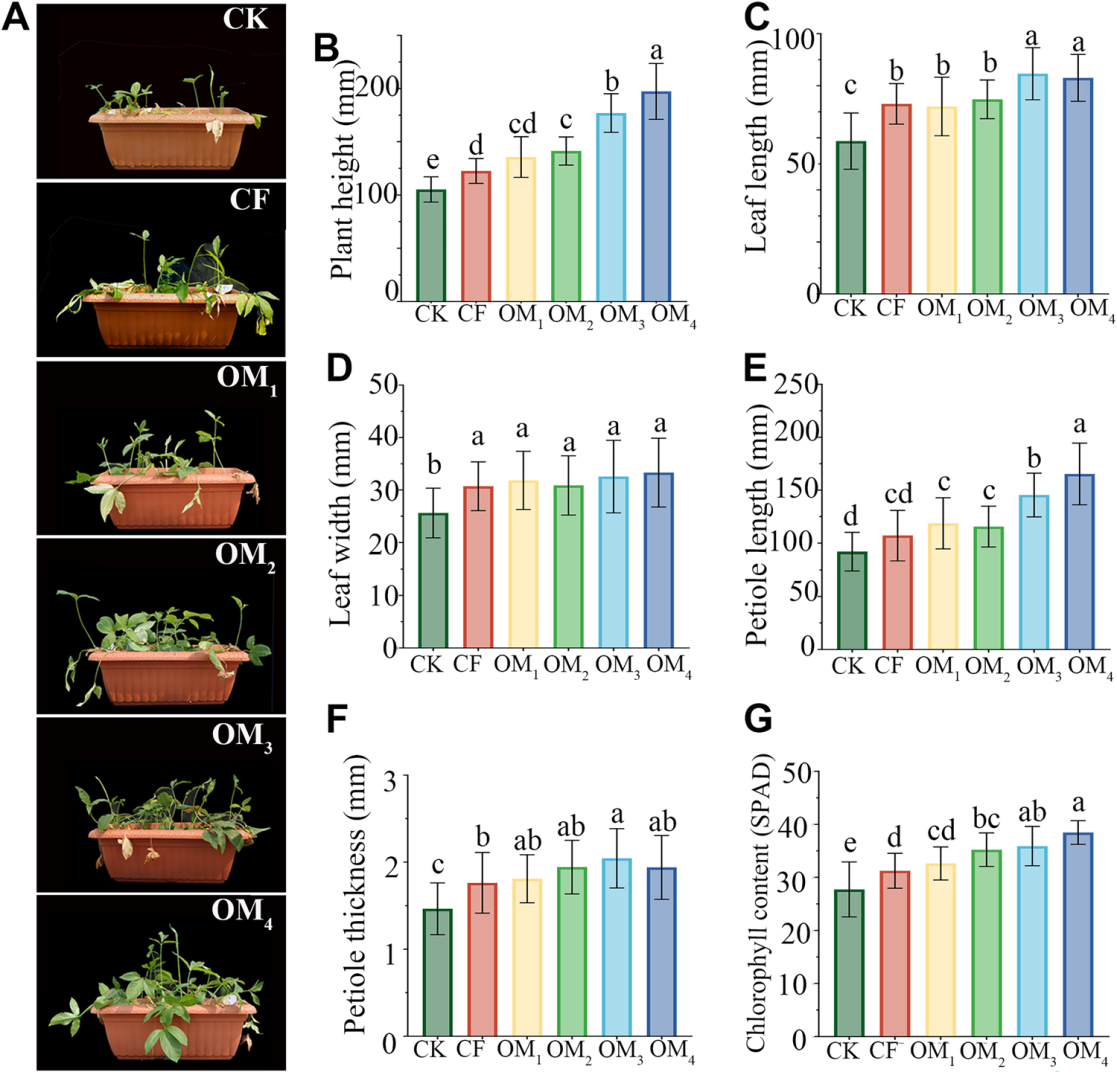
*Pinellia ternata* growth under different fertilizer treatments. Treatments included control check (CK), chemical fertilizer only (CF), and a series of increasing proportions of replacement of chemical with organic fertilizer (OM_1–4_) (n = 5). (**A**) Images of the growth condition of *P. ternata* in pots. (**B**-**G**) The mean values per pot of the following variables: (**B**) plant height, (**C**) leaf length (**D**) leaf width, (**E**) petiole length, (**F**) petiole thickness, (**G**) chlorophyll content. The chlorophyll content was indicated by the SPAD value of the instrument reading. The higher the SPAD value indicates, the higher the chlorophyll content. Columns represent are the means ± SDs for five biological replicates. Treatments with the same letter are not significantly different from each other at an alpha level of 0.05 (LSD post hoc test).

During the seedling stage, *P. ternata* growth in different treatments varied greatly (Fig. 1). Plant height increased with an increased organic fertilizer replacement ratio. The OM_4_ group had the greatest height at 197.36 mm, which is 1.88 times the height in the CK treatment (Fig. 1B). Leaf length also varied among treatments, with OM_3_ > OM_4_ > OM_2_ > CF > OM_1_ > CK. The OM_3_ and OM_4_ treatments had significantly greater leaf lengths when compared to CK and CF (*P* < 0.05, Fig. 1C). Every fertilization treatment had significantly greater leaf width than the CK treatment, though there were no significant differences among OF treatments (Fig. 1D). There was a dose–response relationship between the application rate of organic fertilizer and the petiole length and the petiole thickness, which were greatest in OM_4_ and OM_3_, respectively (Fig. 1E,F). Relative chlorophyll content increased with greater OF, reaching above 35 in OM_2_ (Fig. 1G), while the relative chlorophyll content of the CK and CF treatments was 27.73 and 31.26, respectively. In summary, compared to not applying fertilizers (CK), applying fertilizer increased the SPAD value and improved *P. ternata* growth. Plant growth measurements at high proportions OF were significantly greater than at low proportions.

## Replacing chemical fertilizers with organic fertilizers enhances *P. ternata* yield without compromising quality

**Figure 2:**
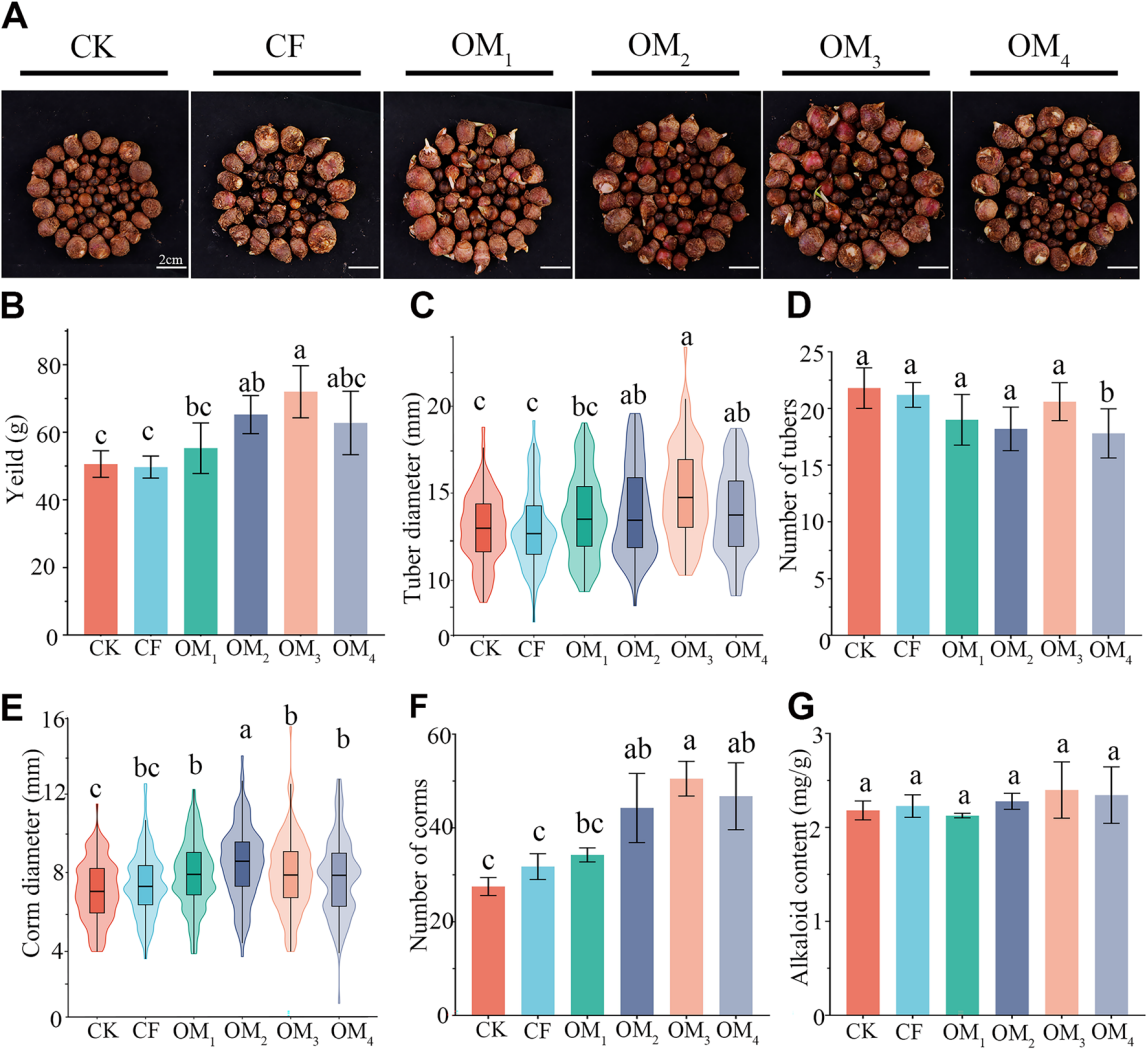
*Pinellia ternata* yield and quality under different fertilizer treatments. Treatments included control (CK), chemical fertilizer only (CF), and a series of increasing proportions of replacement of chemical with organic fertilizer (OM_1–4_). (**A**) The images of harvested *P. ternata* tubers and corms, scale bars: 2 cm. (**B**-**G**) The mean values per pot of the following variables: (**B**) yield, (**C**) tuber diameter, (**D**) number of tubers, (**E**) corm diameter, (**F**) number of corms, (**G**) alkaloid content. (**B**, **D**, **F**, **G**) Columns represent the means ± SDs for five biological replicates. (**C**, **E**) The box plot and Kernel density map show the median, quartile, maximum and minimum values and the distribution of the five biological replicates. Treatments with the same letter are not significantly different from each other at an alpha level of 0.05 (LSD post hoc test).

Compared with CK, the yield of OM_1_, OM_2_, OM_3_, and OM_4_ treatments increased by 9.29%, 28.98%, 42.35%, and 24.06%, respectively, of which OM_2_ and OM_3_ were significantly different (*P* < 0.05; Fig. 2A, 2B). Meanwhile, CF had a yield similar to the CK and was not significantly different (Fig. 2B). To further analyze yield composition, tuber diameter (TD), number of tubers (TN), corm diameter (CD), and number of corms (CN) were measured. OM_3_ had the largest mean TD of 14.65 mm, followed by OM_4_ (13.93 mm) and OM_2_ (13.89 mm) (Fig. 2C). There was little difference in TN among different treatments. However, there was a slight but significant decrease in TN between OM_4_ and the CK treatment (Fig. 2D). The mean CD of the OF treatments was significantly higher than CK and CF, with OM_2_ having the highest value of 8.40 mm (Fig. 2E). There was a positive correlation between mean CN and the amount of organic fertilizer applied, with CK, CF, OM_1_, OM_2_, OM_3_, and OM_4_ having 29, 31, 33, 47, 49, and 47 per pot, respectively (Fig. 2F).

### OF promoted *P. ternata* growth via changes in the "plant hormone signal transduction" and "starch and sucrose metabolism" associated genes

The DEGs of CK versus CF, CK versus OM_3_, and CF versus OM_3_ numbered 253, 987, and 1276, respectively. These DEG numbers indicate that OM_3_ treatment has the largest impact on *P. ternata*. In addition, the number of upregulated genes is much greater than that of downregulated genes (Supplemental Fig. 1). Therefore, we focused on the DEGs of CK versus OM_3_ and CF versus OM_3_ for in-depth analysis.

**Figure 3:**
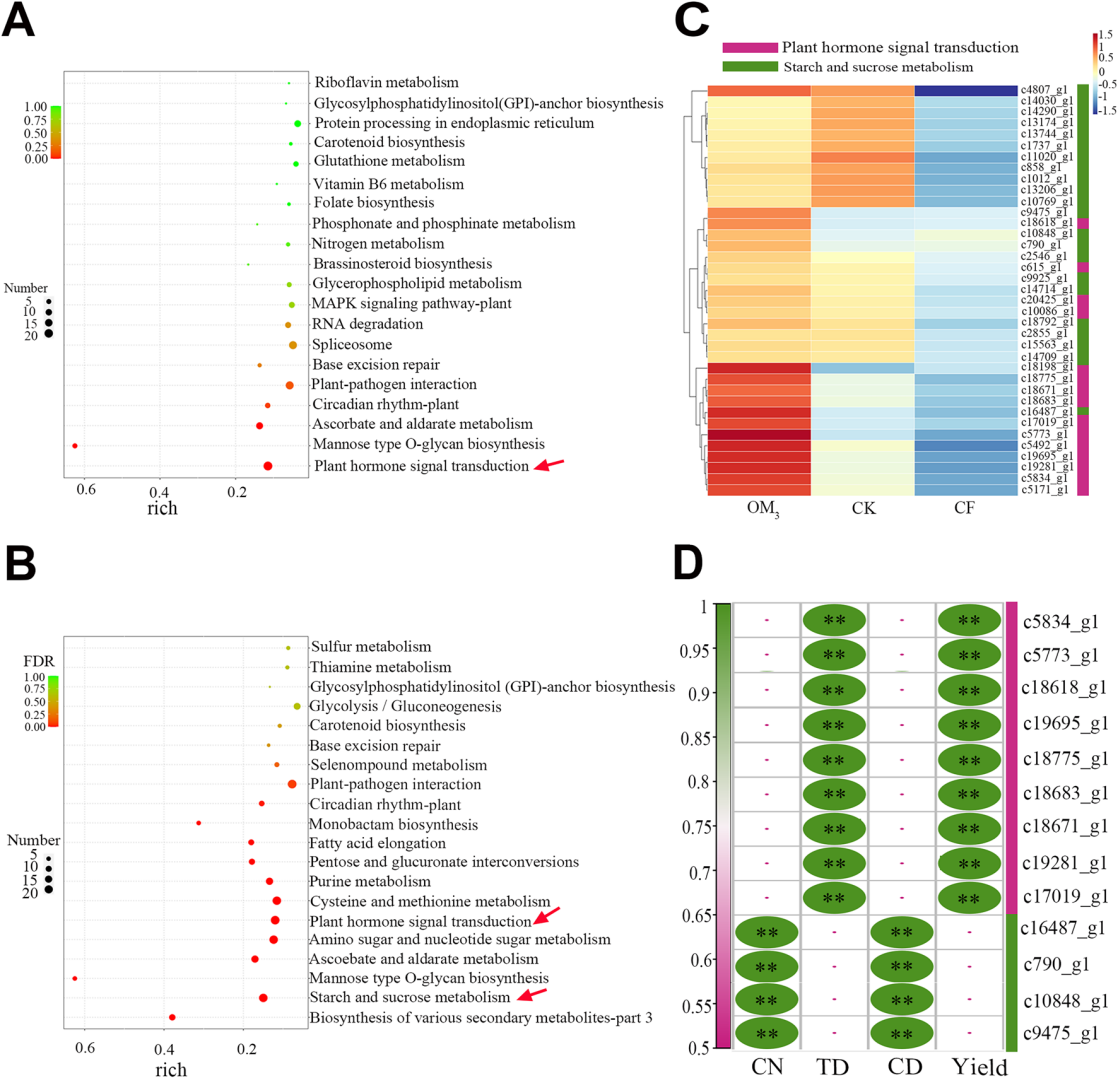
RNA-seq analysis of the *Pinellia ternata* under different fertilizer treatments. Treatments included control (CK), chemical fertilizer only (CF), and 75% replacement of chemical with organic fertilizer (OM_3_). (**A**) Top 20 KEGG pathway enrichment analysis of DEGs of CK versus OM_3_. (**B**) Top 20 KEGG pathway enrichment analysis of DEGs of CF versus OM_3_. (**A**, **B**) The colors are shaded according to the Q-value level, as shown in the color bars gradually from low (red) to high (green); the size of the circle indicates the number of DEGs from small (fewer) to large (greater). (**C**) The expression of key DEGs involved in "Plant hormone signal transduction" and "Starch and sucrose metabolism" pathways. (D) Correlation analysis of key DEGs with yield and yield factors (**P < 0.01;- = *P* > 0.05).

For CK versus OM_3_, the greatest number of DEGs and the most enriched pathways were annotated as "plant hormone signal transduction" (Fig. 3A). In addition, for CF versus OM_3_ "biosynthesis of various secondary metabolites-part3", "starch and sucrose metabolism", "mannose type O-glycan biosynthesis", "aseoebate and aldarate metabolism", "amion sugar and nucleotide sugar metabolism", and "plant hormone signal transduction" were the annotation categories with the most significantly enriched genes (Fig. 3B). Specifically, some genes related to auxin, cytokinin, gibberellin, and brassinosteroid annotated as "plant hormone signal transduction" were upregulated, which may promote plant growth, cell enlargement, and cell division (Supplementary Fig. 2). The 21 DEGs found in "starch and sucrose metabolism" were all upregulated, indicating that the primary metabolism of plants was more active after treatment with organic fertilizers (Fig. 3C, Supplemental Fig. 3). Furthermore, Spearman's rank correlation was used to associate the DEGs expression level with yield and yield composition factors (TD, CD, CN), for which 13 DEGs were labeled as positively correlated (r = 0.6, *P* < 0.05; Fig. 3D). There were 4 DEGs annotated to "starch and sucrose metabolism" and 9 DEGs annotated to "plant hormone signal transduction" that were correlated with TD and yield (Fig. 3D).

### Replacing chemical fertilizers with organic fertilizers change soil enzyme activity

**Figure 4:**
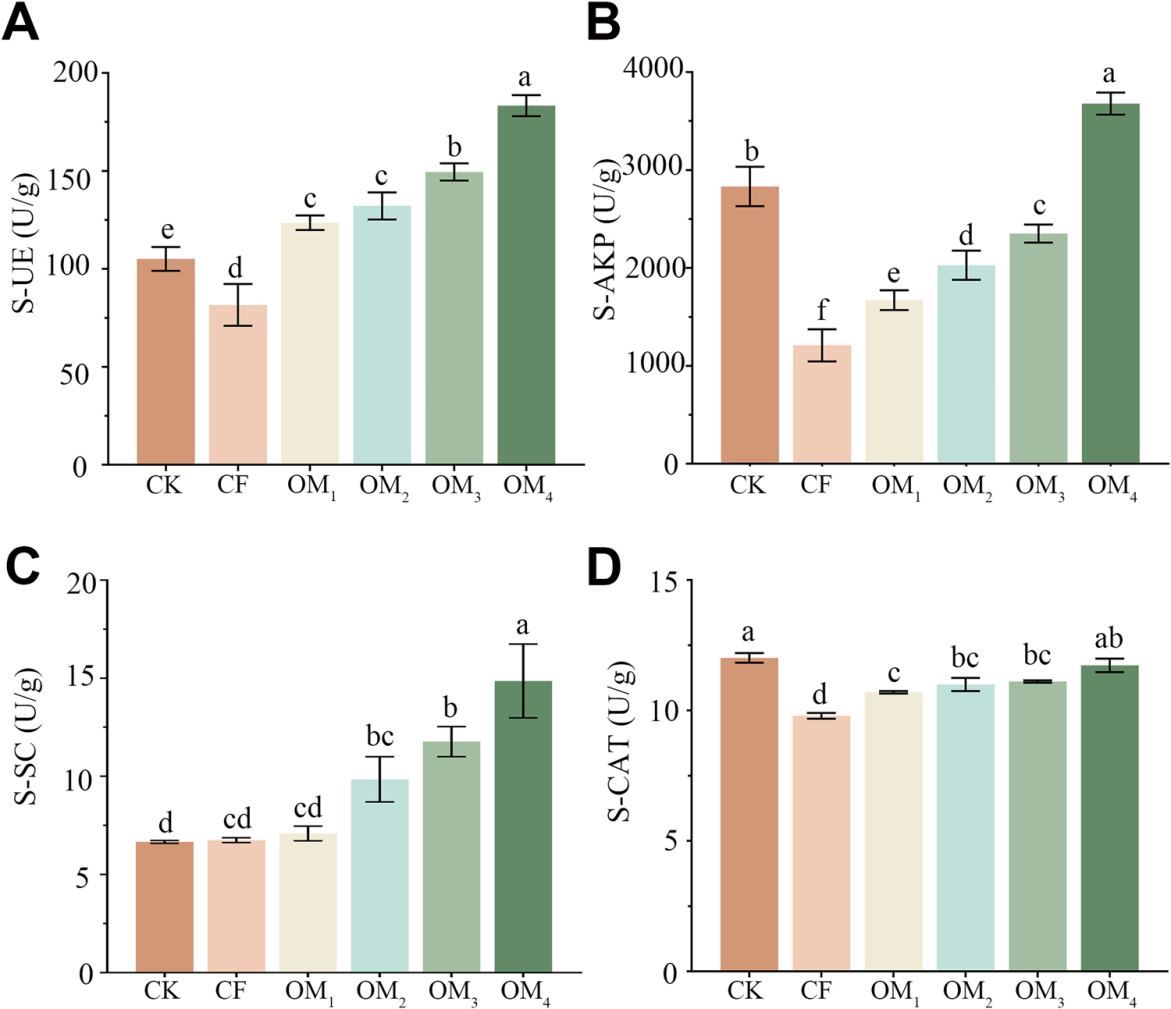
Soil enzyme activity under different fertilizer treatments. Treatments included control (CK), chemical fertilizer only (CF), and a series of chemical fertilizer to organic fertilizer ratios (OM_1–4_). (**A**) Soil urease (S-UE) activities. (**B**) Soil alkaline phosphatase (S-AKP) activities. (**C**) Soil sucrase (S-SC) activities. (**D**) Soil catalase (S-CAT) activities. Columns represent the means ± SDs for five biological replicates. Treatments with the same letter are not significantly different from each other at an alpha level of 0.05 (LSD post hoc test).

The mean S-UE activity in the CF treatment was 81.62 U/g, which was lower than that in the control group CK (105.08 U/g). The mean S-UE of OF treatments was significantly higher than that of CK and CF treatments (Fig. 4A). The mean S-AKP activity of OM_4_ was the highest (3678.19 U/g) among all treatments and was three times that of the CF treatment (Fig. 4B). For mean S-SC activity, the CK treatment had the lowest activity with 6.66 U/g, and CF or OM_1_ treatments did not enhance the activity. However, when the substitution rate reached 50% (OM_2_), the enzyme activity was significantly greater than the CK treatment (Fig. 4C). In contrast, mean S-CAT activity was lower under the CF and OF treatments as compared to the CK treatment, but the differences among treatment groups were smaller compared to other enzymes assays (Fig. 4D). In general, there was a significant dose–effect relationship between soil enzyme activity and the organic fertilizers replacement ratio. Compared with CK and CF, organic fertilizers significantly increased S-UE activity, which catalyzes urea hydrolysis, and S-SC, which decomposes sucrose.

### Organic fertilizers in place of chemical fertilizers change soil microbiome community structure

**Figure 5:**
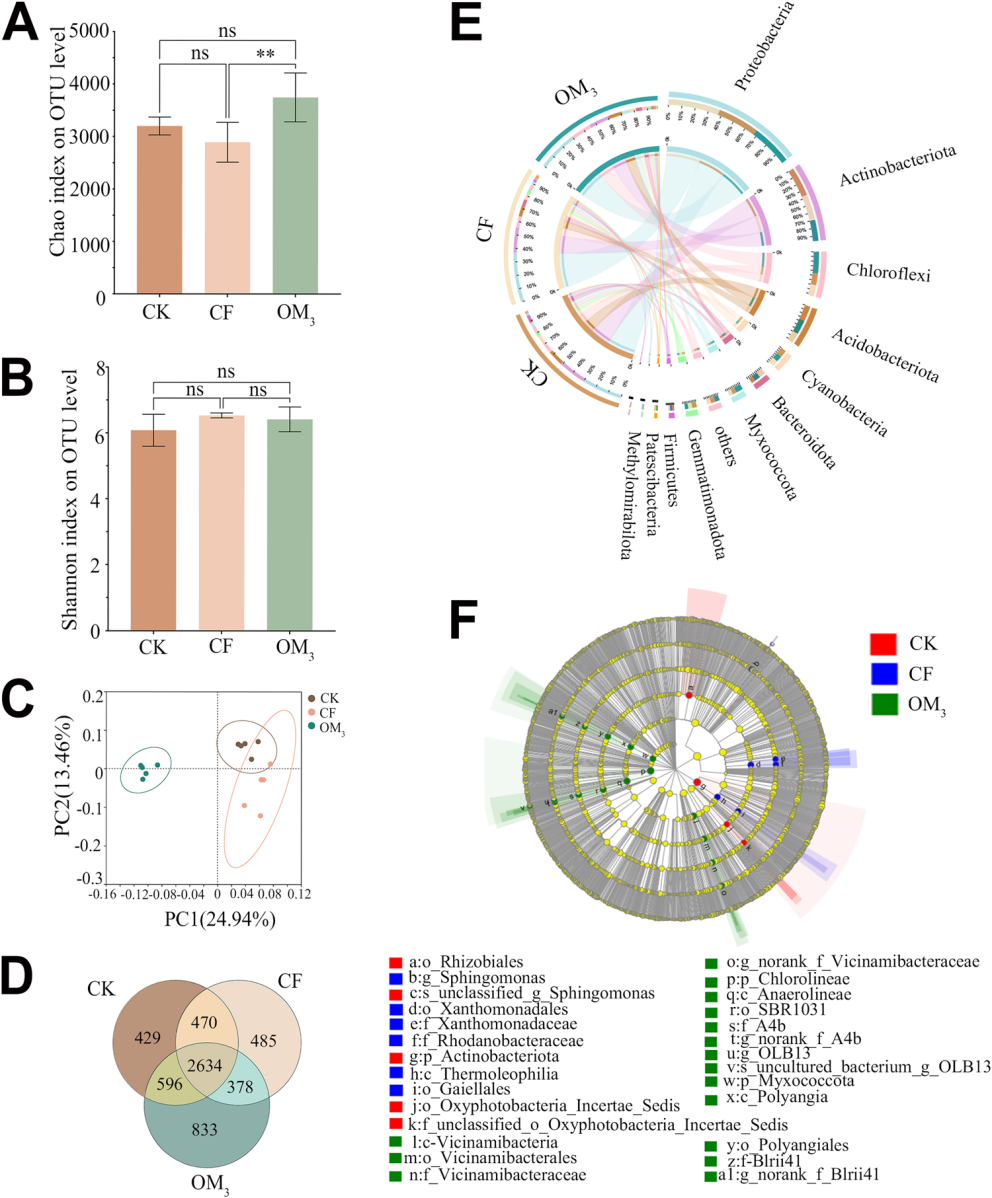
Analysis of microbial diversity in different fertilizer treatments. Treatments included control (CK), chemical fertilizer only (CF), and 75% replacement of chemical with organic fertilizer (OM_3_). (**A**) Chao index in α diversity analysis. (**B**) Shannon index in α diversity analysis. (**C**) Principal coordinate analysis (PCoA) on OTU level. The individual samples are color coded to indicate CK, CF and OM_3_. The plot was analyzed and generated based on the Bray–Curtis distances. (**D**) Venn diagram depicting the number of shared and specific OTUs between treatments. (**E**) Circos graph. The left semicircle represents the phyla composition of each group. The right semicircle indicates the distribution of each phylum in the different groups. (**F**) LEfSe multilevel species difference discriminant analysis (LSD > 4).

The diversity analysis of soil bacteria revealed the microbial changes in *P. ternata* root growth area after organic fertilizers application. The coverage curve and Shannon curve tended to flatten as the sequencing depth deepened, which indicated that they were sufficient to meet the requirements of subsequent data analysis (Supplemental Fig. 4). After Illumina sequencing and quality-filtering, 730,005 sequences were generated, and these sequences were clustered into 5156 OTUs with a 97% sequence similarity cut-off. For α-diversity analysis, the chao index of OM_3_ was 1.17 and 1.29 times greater than that of CK and CF, with CF and OM_3_ having a significant difference (*P* < 0.01; Fig. 5A). The Shannon index showed a slight increase in the OM_3_ treatment compared to other treatments, but it was not significant (Fig. 5B). These results showed that organic fertilizers had little effect on the species diversity of soil bacteria, though they increased the abundance of soil microorganisms. Furthermore, a principal coordinate analysis (PCoA), based on the Bray–Curtis algorithm, was used to estimate overall structural differences in bacterial communities and indicated that CK and CF are clustered together, while OM_3_ is clustered separately (R = 0.8396, *P* = 0.001; Fig. 5C).

The specific composition of microbial communities after different fertilizer treatments was analyzed. While most species were present in all treatments, 429, 485, and 833 unique OTUs were present in the CK, CF, and OM_3_ treatments, respectively (Fig. 5D). Proteobacteria (30–38%) has the highest abundance, followed by Actinobacteriota (16–22%), Chloroflexi (8.6–16%), and Acidobacteriota (7.7–12%) at the phylum level. It is worth noting that the abundance of Chloroflexi and Myxococcota in the OM_3_ treatment is significantly higher compared to the CK and CF treatments (Fig. 5E). Additionally, the LEfSe algorithm was used to find the core microorganisms that lead to significant differences in communities (LDA > 4, *P* < 0.05; Fig. 5F). On the class level, *Polyangia*, *Anaerolineae*, *Vicinamibacteria* (members of Chloroflexi, Myxococcota and Acidobacteriota) were enriched in the OM_3_ treatment. Specifically, the core microorganisms in OM_3_ were *OLB13*, *Vicinamibacteraceae*, and *Blrii41*.

A redundancy analysis (RDA) was used to evaluate the influences of environmental factors on the soil bacterial community composition (Supplemental Fig. 5). The first two axes of RDA explained 30.36% and 21.25% of the total variation in the soil bacterial data, respectively. Furthermore, the OM_3_ treatment was positively correlated with soil pH, SOM, S-UE, and available Zn.

### The enriched microbial community after OF enhanced soil nitrogen cycle

**Figure 6:**
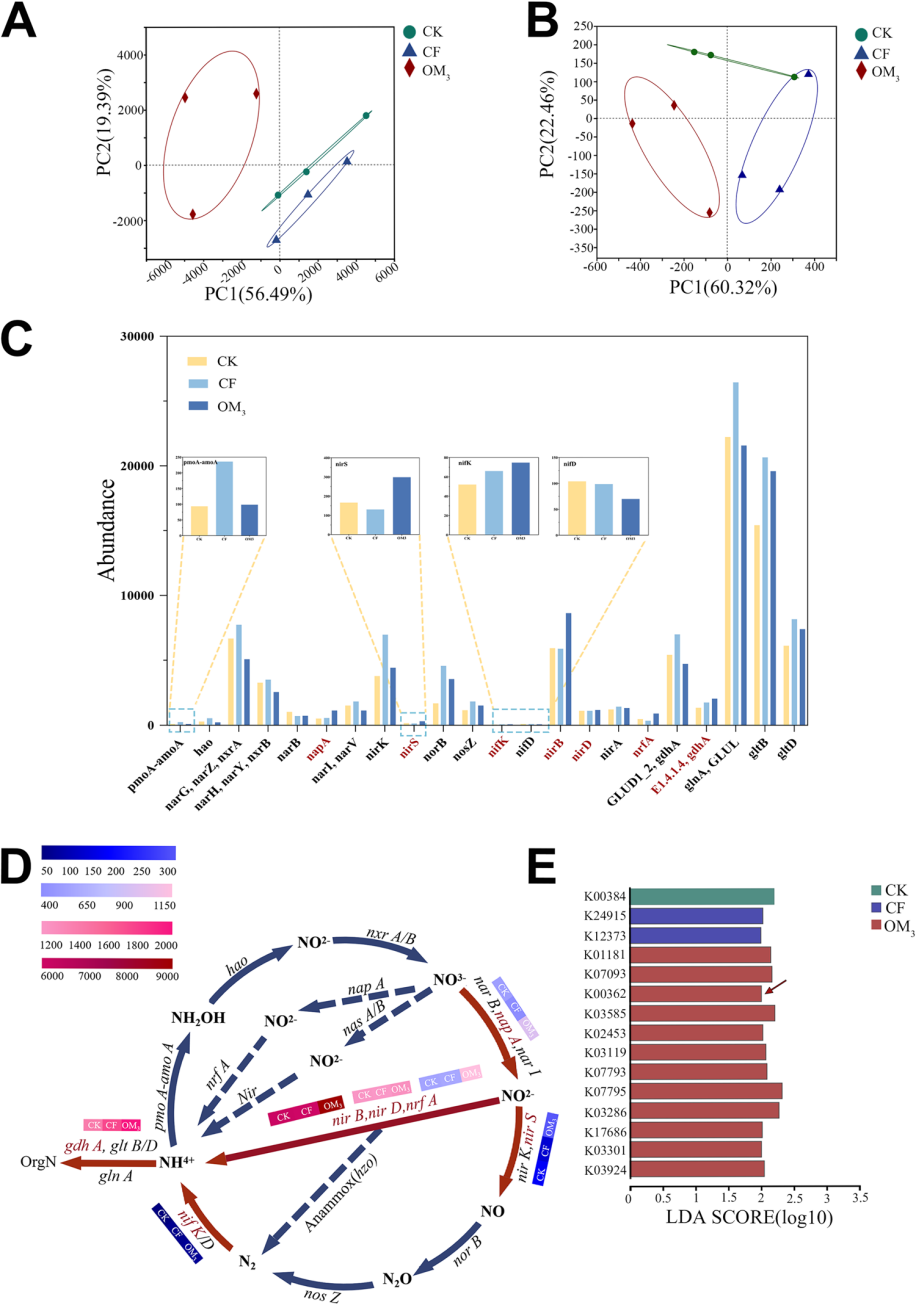
Analysis of soil microbial function under different fertilizer treatments. Treatments included control (CK), chemical fertilizer only (CF), and 75% replacement of chemical with organic fertilizer (OM_3_). (**A**) Principal coordinate analysis (PCoA) of the microbials at KO function level based on the Bray–Curtis distances. (**B**) Principal coordinate analysis (PCoA) of nitrogen cycle genes at KO function level based on the Bray–Curtis distances. (**C**) Soil nitrogen cycle gene relative abundance in different fertilizer treatments. (**D**) Diagram of the microorganism nitrogen cycle pathways in soil. (The red line indicates that genes involved in the process are highly expressed in the OM_3_ group. The blue lines indicate that genes involved in the process are not highly expressed in the OM_3_ group.) (**E**) LEfSe linear discriminant analysis at KO function level.

The soil microorganism community detected in this study is composed of bacteria, eukaryota, archaea, viruses, and unclassified bacteria, with bacteria accounting for 88.60% of the total OTUs (Supplemental. Figure 6). Principal-coordinate analysis (PCoA) showed that the community from the OM_3_ treatment was separated from CK and CF on the PC1 axis, confirming that organic fertilizers changed soil microorganism function (Fig. 6A). Considering that *OLB13*, *Vicinamibacteraceae*, and *Blrii41* were enriched in the OM_3_ treatment and can degrade organic matter and participate in the nitrogen cycle, a gene set relating to soil nitrogen cycle was established using these taxa. The results show that CK, CF, and OM_3_ have significant differences in soil nitrogen cycle functions (Fig. 6B). A total of 22 microbial functional genes were labeled as belonging to the nitrogen cycle pathway (Supplementary Fig. 7), with *nap A*, *nir S*, *nif K*, *nir B*, *nir D*, *nrf A*, and *gdh A* genes being significantly more expressed in OM_3_ (Fig. 6C). It is well known that the nitrogen metabolism pathway is divided into four processes. The highly expressed genes in OM_3_ are mainly involved in converting N-NO_3_^–^ to N-NH_4_^+^ (Fig. 6D). In addition, further LEfSe analysis found that *nir B* (K00362) involved in the nitrogen cycle is a key indicator gene that leads to large differences in soil functions (Fig. 6E).

## Discussion

Since fertility is based on a lot of different biotic and abiotic factors, applying organic fertilizers and reducing chemical fertilizers is an economically feasible and environmentally friendly way for sustainable agricultural development^11,54^. In this study, we explored the feasibility of OF in *P. ternata* cultivation. Our results indicated that this practice significantly enhanced the growth of *P. ternata* (Fig. 1) and increased the yield by increasing the number of corms and the expansion of tubers (Fig. 2). All OF treatments had at least some positive effects on growth and yield. However, the yield of *P. ternata* peaked at the replacement proportion of 75%, with a 42.35% and 44.93% higher yield than CK (control) and CF (chemical fertilizers), respectively (Fig. 2). The alkaloids present in *P. ternata* exhibit therapeutic efficacy against respiratory diseases. They are considered as one of the significant pharmacodynamic constituents^34^. In our study, there was no significant difference in alkaloid content among treatments. The findings suggested that through specific proportions of OF, the goal of increasing *P. ternata* yield by increasing TD, CD, and CN can be achieved without sacrificing quality. These results indicate that the tested fertilization regime is of practical importance to growers and may be profitable to implement in ecological plantings of *P. ternata*. This regime replaces 75% of standard applications of chemical fertilizers with organic fertilizer, representing a replacement of 550.42 kg/ha organic fertilizer and 148.01 kg/ha chemical fertilizers.

The mechanism behind the enhanced plant growth observed after the application of organic fertilizers was further studied. Soil physicochemical properties are considered indicators of soil quality, the ability of soil to maintain environmental quality and promote plant productivity^55^. Obviously, the soil organic matter (SOM) content increased significantly when organic fertilizers were applied (Supplemental Table 1). Previous studies have shown that SOM can affect the biosynthetic pathways of hormones in young leaves and roots of plants and can contribute to the accumulation of growth-promoting hormones^56^. The transcriptome analysis results in the current study agree with these previous findings (Fig. 3A,B). Some mineral elements in the soil also increased (Supplemental Table 1), of which the change in zinc deserves special attention. Zinc is an essential element for chlorophyll biosynthesis, and its accumulation may promote the biosynthesis of chlorophyll, thereby enhancing the photosynthetic capacity of plants^57^. It can also increase aldolase activity and decrease amylase activity, thereby promoting starch accumulation^58^. These results indicate that organic fertilizers accelerate the formation and expansion of corms by promoting starch and sucrose metabolism, as well as promote plant hormone signal transduction to encourage tuber enlargement, ultimately improving the yield of *P. ternata*.

Furthermore, soil enzyme activity can reflect the nutrient cycling capability of soil, and the measurement of soil enzyme activity can be used as an indicator of soil fertility^59^. Our results indicate that compared with traditional chemical fertilization, OF can alter the activity of soil enzymes, especially S-UE (Fig. 4). S-UE is the only enzyme that has an important effect on urea conversion, and its enzymatic product, ammonia, is a plant nitrogen source^60^. In short, soil nutrient status was improved, and fertility increased after adding organic fertilizers compared to only chemical fertilizers or control. The increase of the activity of these two enzymes contributed to the increase in plant growth seen in the current study.

It is worth noting that changes in soil physicochemical properties do not directly act on plants but rather affect plants by altering the composition of soil microorganisms, which then affect plants. Microbial diversity analysis found that there were differences in soil microbial community structure between different fertilization treatments, especially between organic fertilizer application and organic fertilizer absence (Fig. 5C). RDA analysis also found that changes in soil factors such as SOM, S-UE, and Zn were associated with the formation of the microbial community structure found in the OM_3_ treatment, which may be the key environmental factors that lead to the favorable development of the soil microbial community after organic fertilizers application (Supplemental Fig. 5). At a deeper level, *OLB1*3 of Chlorolineae, *Blrii41* of Myxococcota, and *Vicinamibacteraceae* of Vicinamibacteria were distinct species in the OM_3_ community (Fig. 5). These microorganisms were previously reported to be associated with the decomposition of organic matter and the soil nitrogen cycle^61–63^. According to previous reports, members of the *Blrii41* clade are often abundant in organic soils^64,65^. The members of the *Vicinamibacteraceae* family can degrade complex organic compounds, such as chitin, which can transform macromolecular organic substances into small-molecule organic substances that are easier for plants to take up^64^. In addition, *OLB13* can reduce nitrate and nitrite, participating in the soil nitrogen cycle^63^. Overall, the microorganisms favored by the application of organic fertilizers are mainly involved in the decomposition of organic matter and the soil nitrogen cycle, which may be closely related to the promotion of plant growth.

Previous studies have found that different fertilization treatments lead to significant differences in soil bacterial communities, and changes in microbial composition often led to corresponding changes in soil function. Metagenomic analysis was used to further explore the differences in microbial function among treatment groups. The results showed that OF significantly impacted nitrogen metabolism in soil (Fig. 6), which is consistent with the function of core microorganisms enriched in the OM_3_ treatment group. Generally, the soil nitrogen cycle includes nitrification, denitrification, nitrogen fixation, assimilation nitrate reduction, dissimilation nitrate reduction, and organic nitrogen metabolism^66^. Among the highly differentially expressed genes in the OM_3_, *nap A*, *nir S*, and *nif K* genes are involved in soil denitrification, while the *nir B*, *nir D*, and *nrf A* genes are involved in the dissimilation nitrate reduction pathway. Jointly, these two sets of genes promote the transformation of soil N-NO_3_^−^ to N-NH_4_^+^ (Fig. 6D), which allows plants to consume less energy than performing the conversion themselves, and *nir B* may be an important driver of denitrification after adding organic fertilizers.

Interestingly, plants require less energy to absorb and assimilate ammonium nitrogen, making it easier to absorb than nitrate nitrogen^13,67^, which may improve the utilization efficiency of soil nutrients by *P. ternata*. Hu et al.^68^ have found that *P. ternata* is a plant that prefers N-NH_4_^+^. When the ratio of N-NH_4_^+^/ N-NO_3_^−^ reaches a ratio of 75:25, the chlorophyll a, carotenoid content, the number of tubers and corms, and total yield were all the highest. Moreover, the total organic acids in *P. ternata* tubers show an upward trend with an increasing N-NH_4_^+^/ N-NO_3_^−^ ratio^69^. The results from previous multi-year field trials on organic fertilizer management agree with the current study. Previous experiments have shown that the abundance of denitrification genes such as *nirS*, *nosZ*, *nifH*, *nirK* increased under organic fertilizer regimes, thus contributing to higher yields in cotton^70^, corn^71^ and grapes^13^. Following OF applications, we suspect that microorganisms were contributing more easily absorbed nitrogen (ammonia) to the soil environment, and we found that the N content in *P. ternata* leaves was significantly greater than following treatments where this ammonia was not supplied (CK and CF) (Supplemental Fig. 8). Nitrogen is a component of all proteins, as well as various other plant compounds such as chlorophyll, a critical molecule involved in photosynthesis^72^. Generally, a higher nitrogen content corresponds to a faster photosynthetic rate^73^. As a result of higher photosynthesis, it is thought that the efficiency of synthesis and accumulation of primary metabolites will be significantly increased. The results of our transcriptome analysis verified this hypothesis.

The expression of many genes in the "starch and sucrose synthesis" pathway in *P. ternata* tubers was significantly higher in OF-treated plants, and four genes were positively correlated with corm diameter and number in those treatments. We believe this is an important reason for *P. ternata* tuber enlargement and rapid corm propagation. In addition, some plant hormone synthesis genes that can promote growth were upregulated in the OF treatment, such as auxin, cytokinin, and brassinosteroid (Supplement Fig. 2). The ultimate results of these hormones on plants are mainly cell enlargement, plant growth, cell division, shoot initiation, and cell elongation, all of which may also be other key factors in the faster growth of *P. ternata* following organic fertilizer application.

## Conclusion

**Figure 7:**
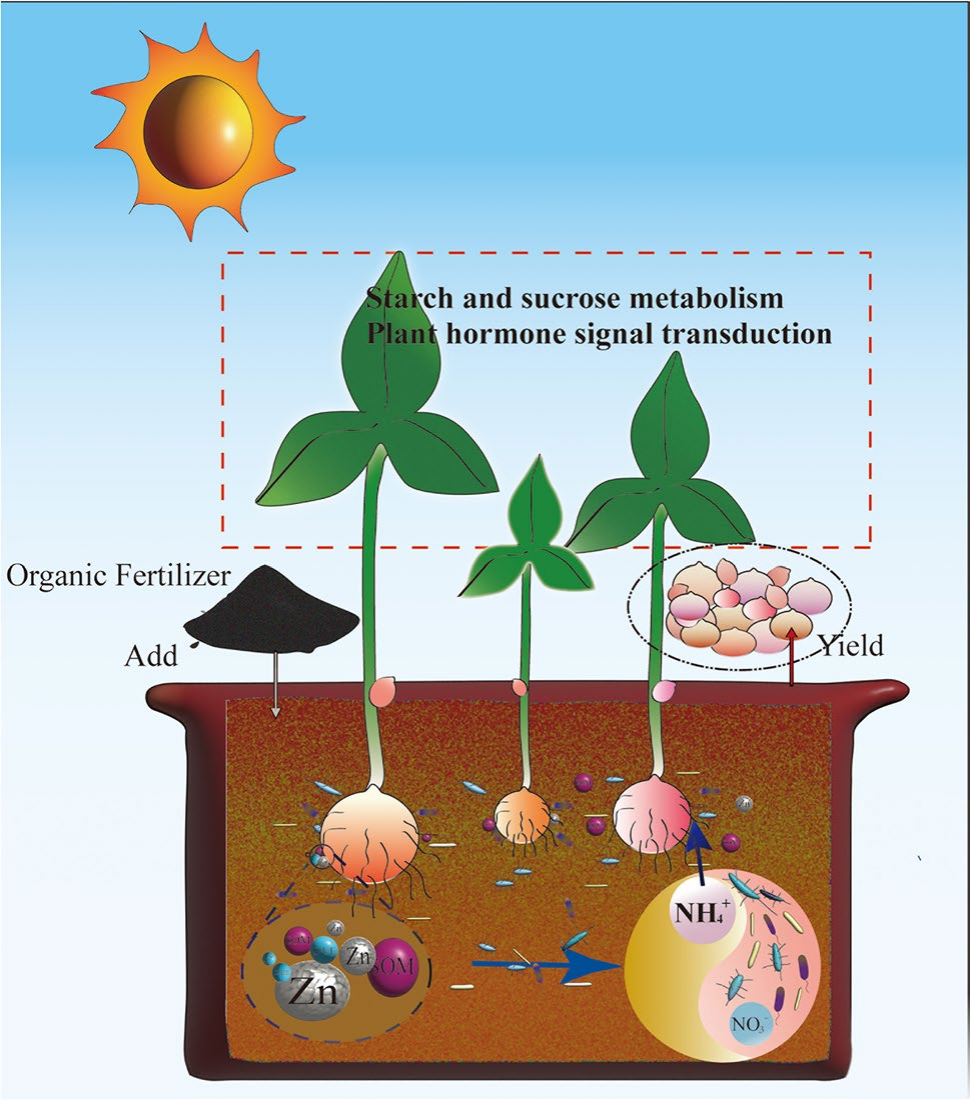
A diagram of the proposed model of the mechanisms that promotes the increase of *Pinellia ternata* yield following OF.

In this study, we combined soil physical and chemical properties, microbial diversity, metagenomics, and plant transcriptomics to systematically demonstrate that the application of organic fertilizer can significantly improve soil environment and nutrient status. Some key environmental factors, such as the increase of SOM, Zn, and S-UE can promote the enrichment of beneficial microorganisms. These microorganisms can convert N-NO_3_^−^ into N-NH_4_^+^, which is more easily absorbed by plants, further promoting the synthesis of growth-promoting hormones, starch, and sucrose in *P. ternata*, thereby achieving greater yield in *P. ternata* (Fig. 7). These results provide positive application prospects for the planting mode of replacing chemical fertilizers with organic fertilizers in agriculture. Furthermore, the microbiome approach used in this article also provide novel insights into the theory that successful fertilization regimes achieve yield increases by recruiting beneficial microorganisms. However, these microorganisms need to be further isolated, screened, and validated for their functions to further development of organic agriculture.

### Supplementary Information

Supplementary Information.

## Data Availability

Raw transcriptomic data have been deposited in the NCBI SRA database (BioProject ID: PRJNA1002642). The raw reads associated with 16S rRNA sequencing were deposited into the NCBI Sequence Read Archive (SRA) database (Accession Number: PRJNA1012746). The metagenome sequences have been deposited in the NCBI database under the accession number PRJNA1015345. All other data are included as supplemental material to this paper.
