# Supplementary material for: Organic fertilizer application promotes the soil nitrogen cycle and plant starch and sucrose metabolism to improve the yield of Pinellia ternata
Source: Sci Rep. 2024 Jun 3;14:12722. doi: 10.1038/s41598-024-63564-0 (PMC11148117; doi:10.1038/s41598-024-63564-0)
Supplement: Supplementary file 1 — Supplementary Information. [file 41598_2024_63564_MOESM1_ESM.docx]

**Organic fertilizer application promotes the soil nitrogen cycle and plant starch and sucrose metabolism to improve the yield of *Pinellia ternata***

**Lu Wei^†a^, Jinxin Li^†a^, Kaili Qu^†a^, Hong Chen^a^, Mingxing Wang^a^, Shuaijie Xia^a^, Huixia Cai^a^, Xien Long^b^, Yuhuan Miao^a^, and Dahui Liu*^a^**

^a^ *Pharmacy Faculty, Hubei University of Chinese Medicine, Wuhan 430065, China*

*^b^ School of Geographic Sciences, Nantong University, Nantong 226019, Jiangsu, China*

* For correspondence (e-mails liudahui@hbtcm.edu.cn).

† These authors contributed equally to this work.

**Supplemental Figures**

**Figure S1**


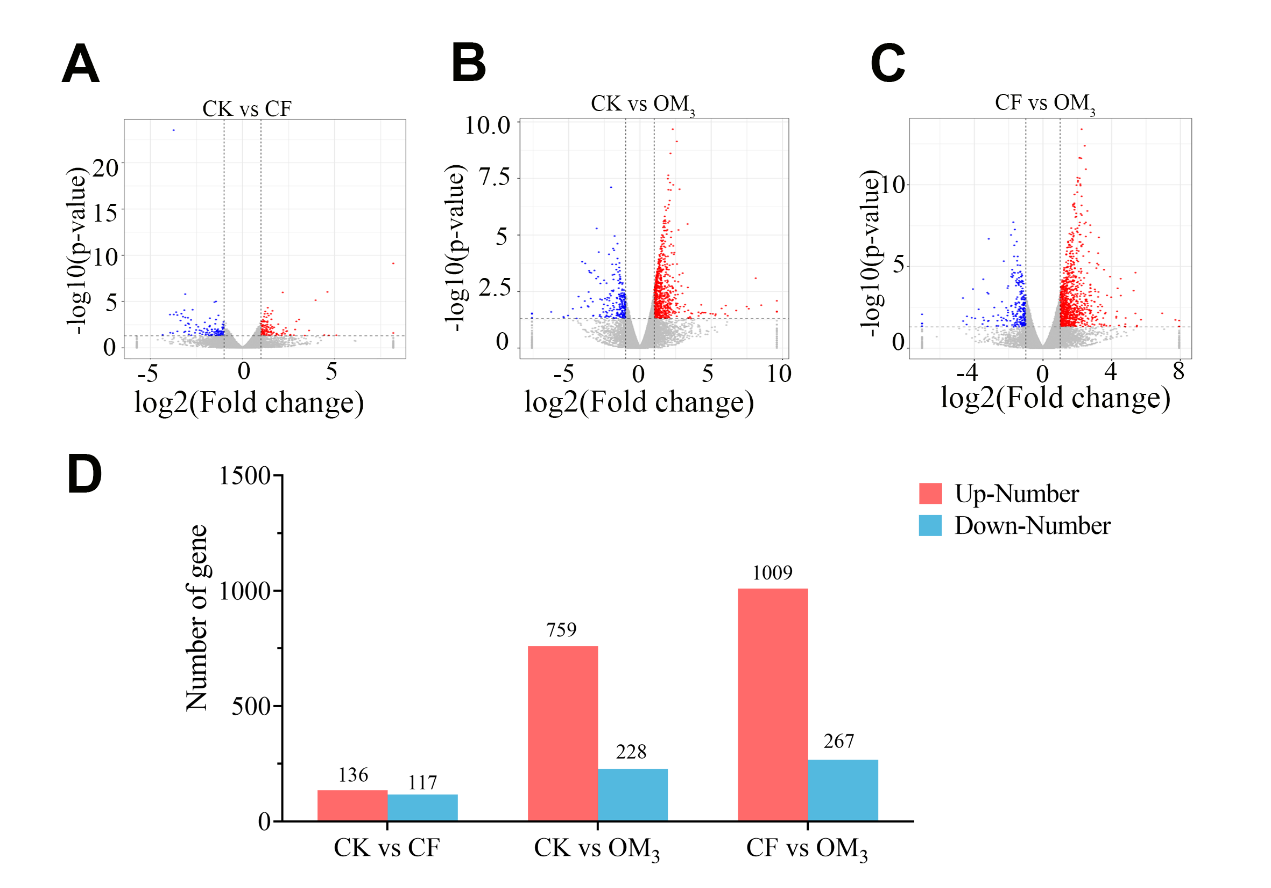


**Figure S1.** **Number of up-regulated and down-regulated genes in the *P. ternata* transcriptome.** (A-C) Volcanic plot of unigenes compared between different fertilizer treatment groups. (D) Number of up-regulated and down-regulated genes of the *P. ternata* compared between different fertilizer treatment groups. Treatments included no fertilizer check (CK), chemical fertilizer only (CF), and 75% replacement of chemical with organic fertilizer (OM_3_).

**Figure S2**

**
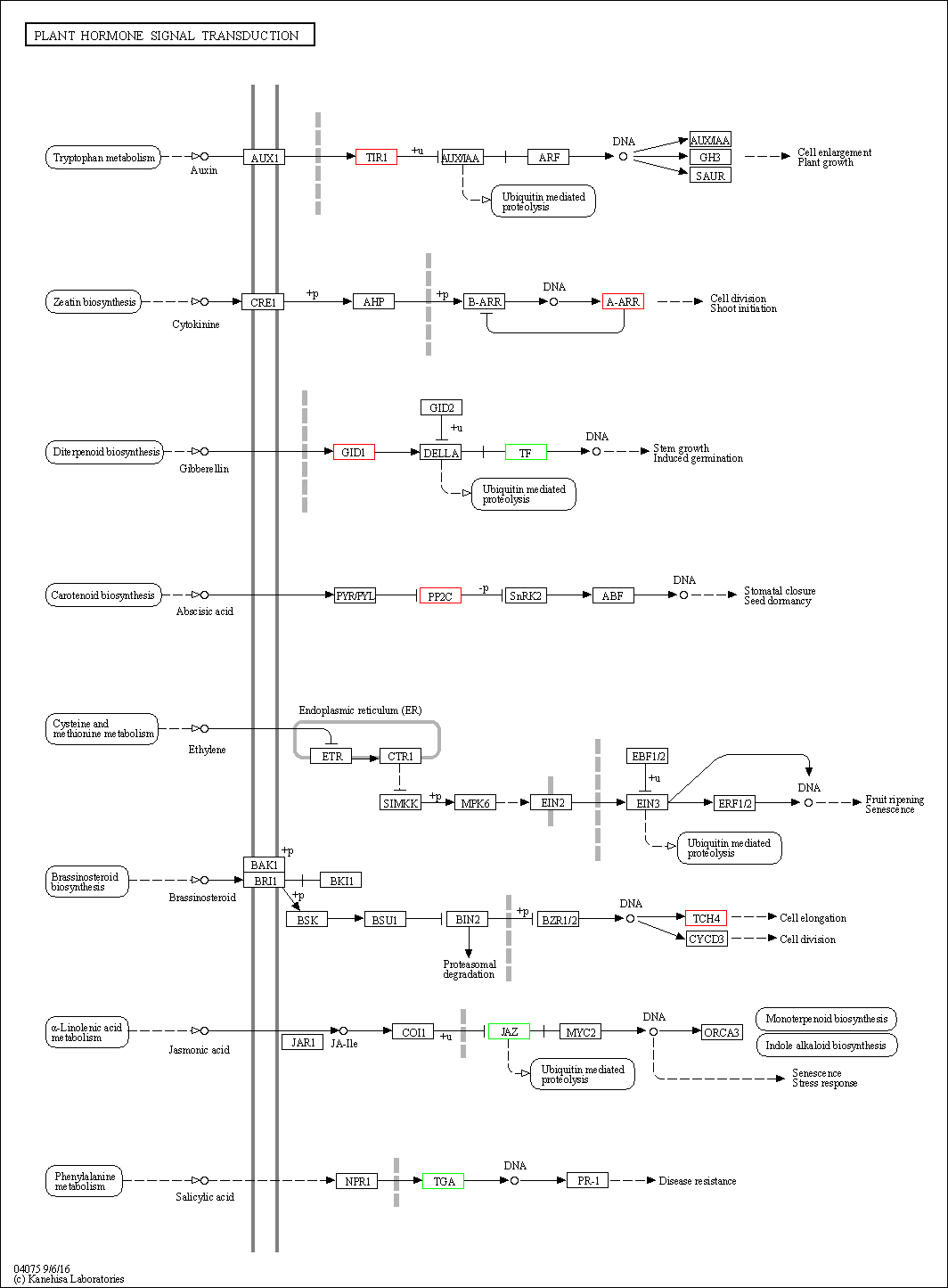
**

**Figure S2**. **The DEGs involved in “plant hormone signal transduction” pathways in *P. ternata* RNA-Seq analysis.**

**Figure S3**

**
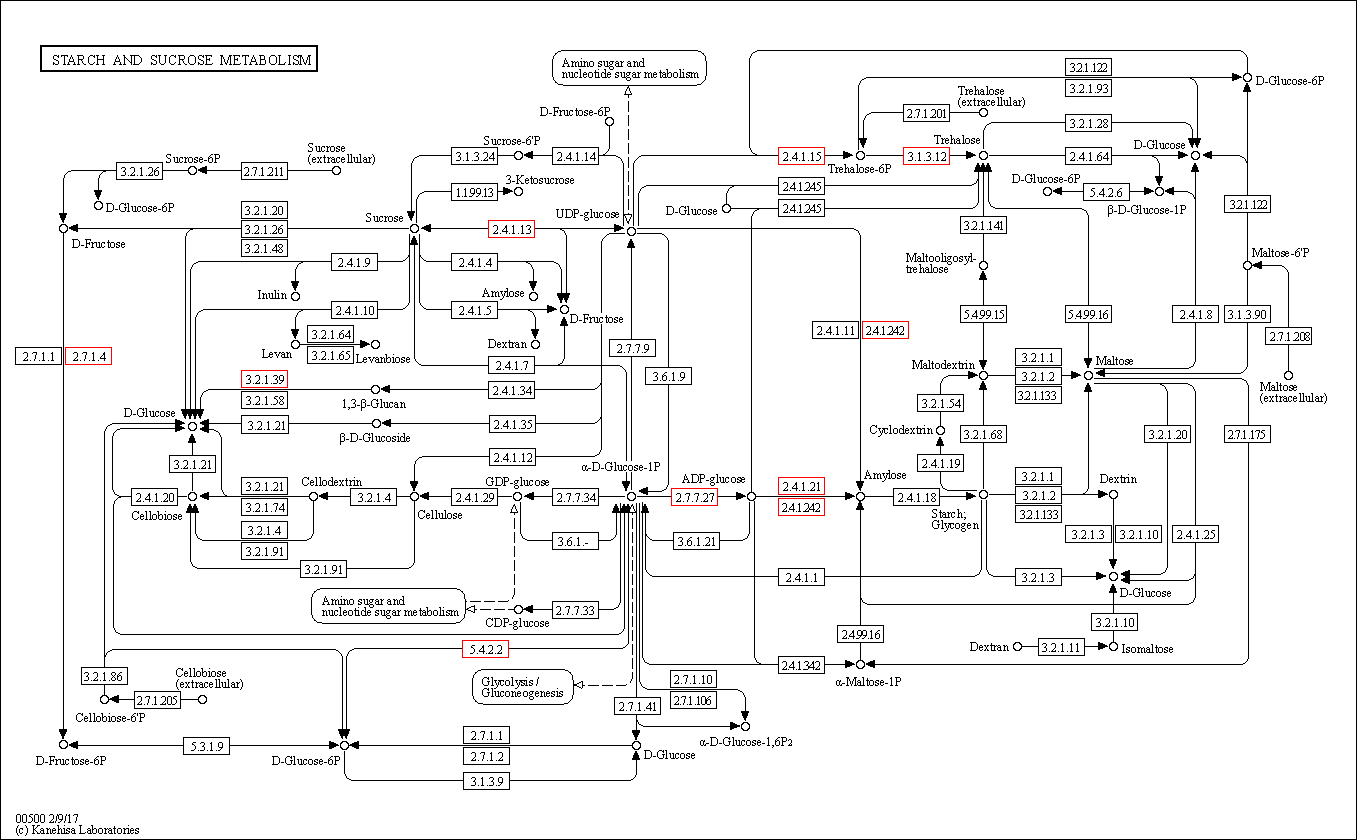
**

**Figure S3**. **The DEGs involved in “starch and sucrose metabolism” pathways in *P. ternata* RNA-Seq analysis.**

**Figure S4**


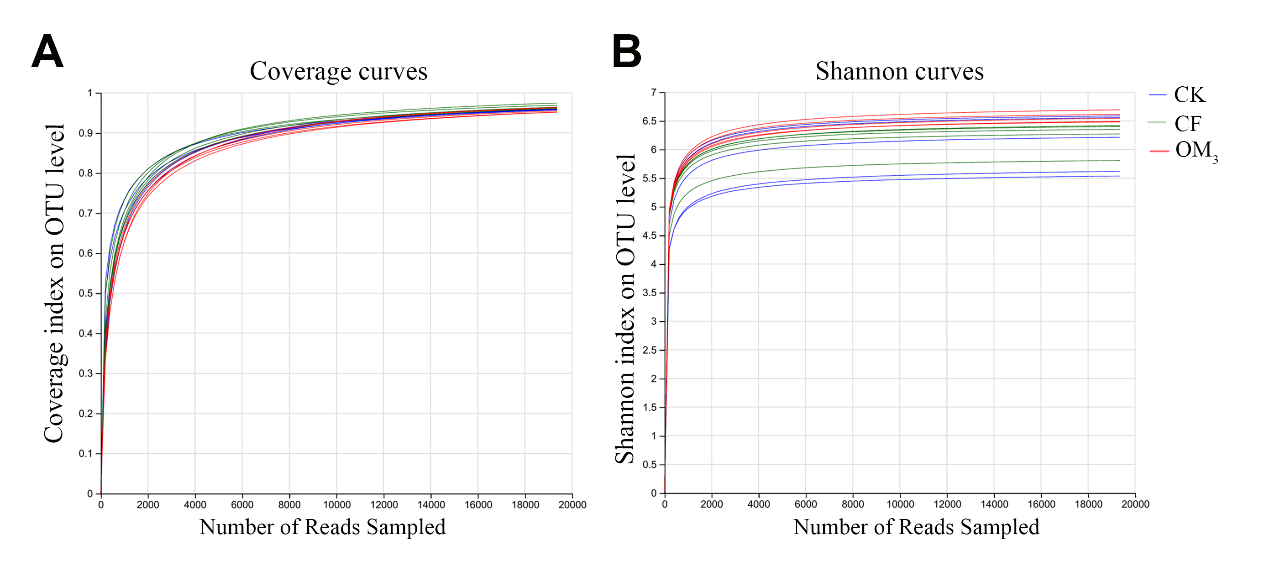


**Figure S4.** **Quality control curve.**The coverage curves (A) and shannon curves(B) of 16S diversity analysis. Treatments included no fertilizer check (CK), chemical fertilizer only (CF), and 75% replacement of chemical with organic fertilizer (OM_3_).

**Figure S5**


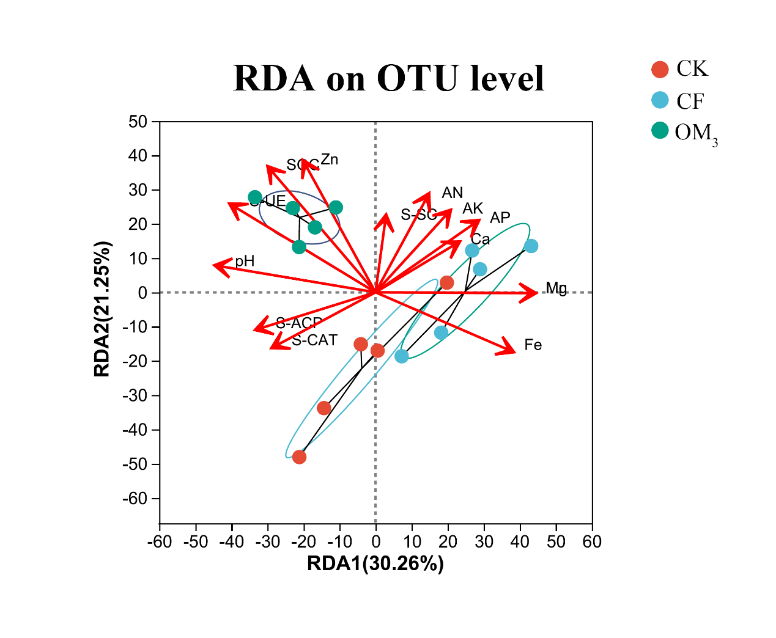


**Figure 5. Redundancy analysis (RDA) of environmental properties and bacteria community on the OTU level.** Treatments included no fertilizer check (CK), chemical fertilizer only (CF), and 75% replacement of chemical with organic fertilizer (OM_3_).

**Figure S6**

**
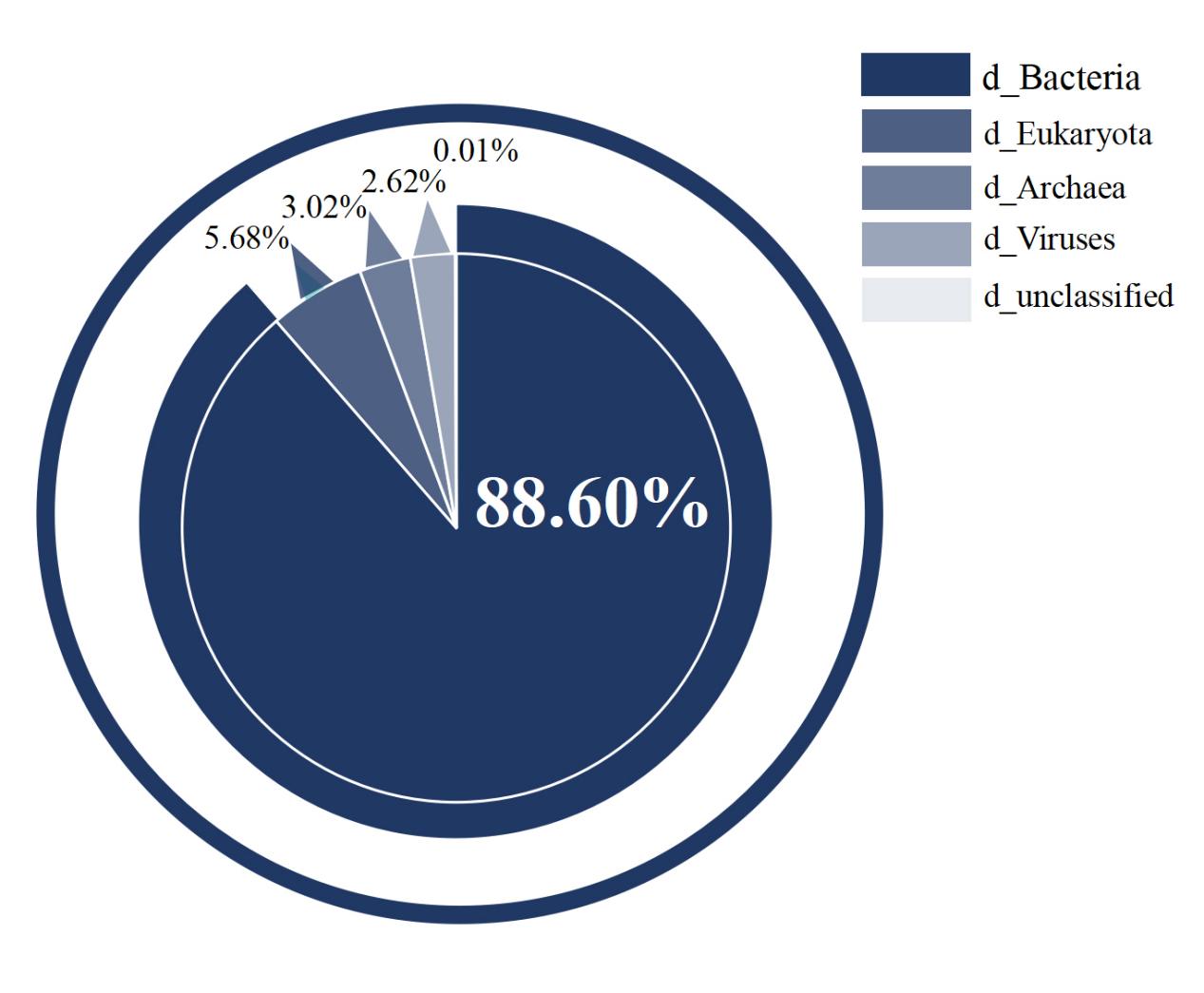
**

**Figure S6.** **Composition and proportion of soil** **microorganisms.**

**Figure S7**


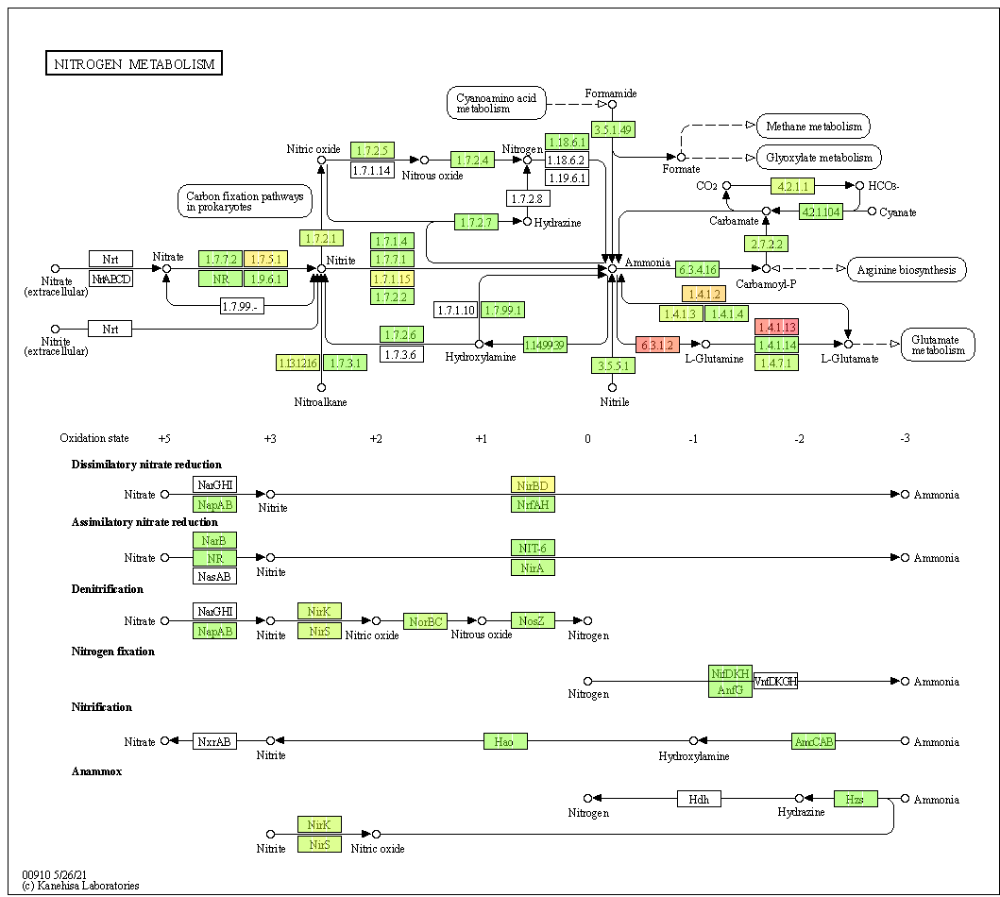


**Figure 7. The DEGs involved in nitrogen metabolism between different group microorganisms.**

**Figure S8**


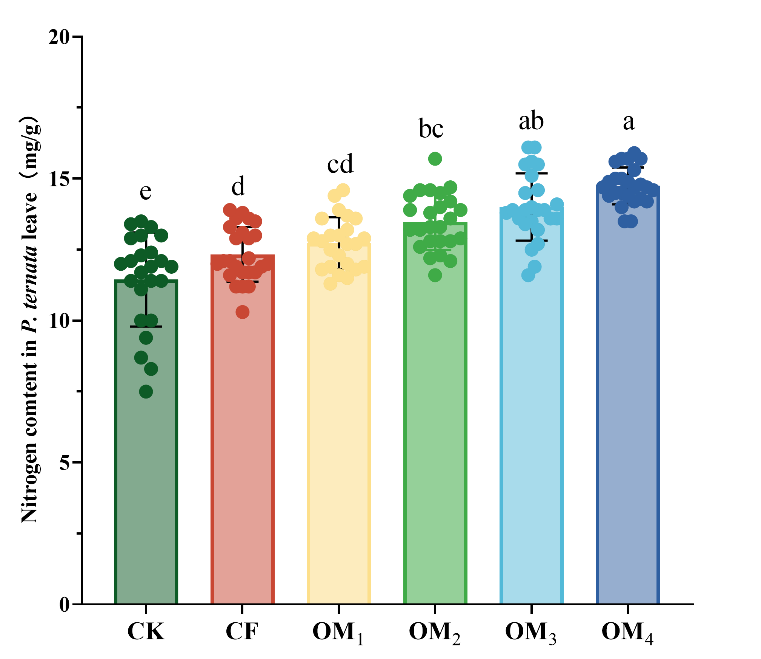


**Figure S8. Nitrogen content in *P. ternata* leaves under different fertilizer treatments.** Treatments included no fertilizer check (CK), chemical fertilizer only (CF), and a series of increasing proportions of replacement of chemical with organic fertilizer (OM_1-4_).

**Table S1.**  Soil properties under different fertilization treatments (mg/kg).

|  | CK | CF | OM_1_ | OM_2_ | OM_3_ | | OM_4_ |
| --- | --- | --- | --- | --- | --- | --- | --- |
| SOM | 3825.01±356.54 | 3876.16±116.99 | 3874.88±252.48 | 4496.94±141.63 | | 5148.41±433.59 | 6128.32±287.37 |
| E-Ca | 240.79±52.72 | 601.48±62.11 | 370.33±83.95 | 287.04±90.13 | | 416.54±62.11 | 222.29±50.64 |
| E-Mg | 1450.75±87.40 | 1809.70±20.53 | 2056.92±45.90 | 1612.55±26.10 | | 1407.44±16.62 | 1106.98±94.79 |
| A-Fe | 17.41±0.94 | 18.15±0.29 | 19.69±0.92 | 17.53±1.43 | | 16.26±0.82 | 15.86±0.29 |
| A-Mn | 46.44±2.79 | 57.92±0.65 | 65.84±1.47 | 51.62±0.84 | | 45.05±0.53 | 35.44±3.03 |
| A-Zn | 1.75±0.02 | 2.14±0.4 | 2.23±0.15 | 2.47±0.05 | | 2.98±0.32 | 3.24±0.15 |

note: CK: no fertilizer, CF: chemical fertilizers, OM_1_-OM_4_: Organic fertilizers replaces chemical fertilizers with different proportions. The total amounts of nutrients of each treatment are equal except CK. The proportion of organic fertilizers replacement was calculated according to the amount of N, P and K in the fertilizers. E-Ca and E-Mg are short for soil exchangeable calcium (Ca) and magnesium (Mg); A-Fe, A-Mn and A-Zn are short for soil available iron (Fe), manganese (Mn), and zinc (Zn).
